# Clinical Basis for Creating an Osseointegrated Neural Interface

**DOI:** 10.3389/fnins.2022.828593

**Published:** 2022-04-12

**Authors:** Alison M. Karczewski, Weifeng Zeng, Lindsay M. Stratchko, Kent N. Bachus, Samuel O. Poore, Aaron M. Dingle

**Affiliations:** ^1^Division of Plastic Surgery, Department of Surgery, University of Wisconsin School of Medicine and Public Health, Madison, WI, United States; ^2^Department of Radiology, University of Wisconsin School of Medicine and Public Health, Madison, WI, United States; ^3^George E. Wahlen Department of Veterans Affairs Medical Center and the Department of Orthopaedics, University of Utah Orthopaedic Center, Salt Lake City, UT, United States

**Keywords:** amputation, neuroprosthetics, osseointegration (OI), peripheral neural interface, osseointegrated neural interface, clinical translation

## Abstract

As technology continues to improve within the neuroprosthetic landscape, there has been a paradigm shift in the approach to amputation and surgical implementation of haptic neural prosthesis for limb restoration. The Osseointegrated Neural Interface (ONI) is a proposed solution involving the transposition of terminal nerves into the medullary canal of long bones. This design combines concepts of neuroma formation and prevention with osseointegration to provide a stable environment for conduction of neural signals for sophisticated prosthetic control. While this concept has previously been explored in animal models, it has yet to be explored in humans. This anatomic study used three upper limb and three lower limb cadavers to assess the clinical feasibility of creating an ONI in humans. Anatomical measurement of the major peripheral nerves- circumference, length, and depth- were performed as they are critical for electrode design and rerouting of the nerves into the long bones. CT imaging was used for morphologic bone evaluation and virtual implantation of two osseointegrated implants were performed to assess the amount of residual medullary space available for housing the neural interfacing hardware. Use of a small stem osseointegrated implant was found to reduce bone removal and provide more intramedullary space than a traditional implant; however, the higher the amputation site, the less medullary space was available regardless of implant type. Thus the stability of the endoprosthesis must be maximized while still maintaining enough residual space for the interface components. The results from this study provide an anatomic basis required for establishing a clinically applicable ONI in humans. They may serve as a guide for surgical implementation of an osseointegrated endoprosthesis with intramedullary electrodes for prosthetic control.

## Introduction

As of 2005 there were a total of 1.6 million individuals in the United States who had experienced a limb loss, with an expected doubling to 3.2 million over the next three decades (Ziegler-Graham et al., 2008). Individuals with amputations frequently experience debilitating functional deficits and painful neuromas that greatly affect quality of life (Varma et al., 2014). In recent years there have been new developments in prosthetic limbs that aim to address these issues from both an engineering and surgical perspective. Technological advancements in biomechanics have allowed for prosthetic limbs to progress from body powered devices that provide singular control, to more innovative neural interfaces that provide intuitive control (Vu et al., 2020a). These new developments seek to address both mechanical issues related to prosthetics as well as sophistication of the motor control system.

With the progression of myoelectric devices to peripheral neural interfaces, there has been a push for a bidirectional closed loop system that integrates both motor and sensory feedback into the prosthetic to provide synergistic control and enhance embodiment for the individual (Tan et al., 2015; Schofield et al., 2020). Initially, artificial, non-invasive methods of incorporating sensory perception were explored, however, these methods lack selectivity and thus more invasive methods with implantable electrodes and neural interfaces have been pursued (Tan et al., 2015; Ghafoor et al., 2017). Among the different types of electrodes, the common understanding is that there is a trade-off between selectivity and stability. Less invasive electrodes are limited in selectivity due to activation of larger afferent populations and cross talk from surrounding muscles and nearby fascicles (Micera et al., 2010). Whereas more invasive electrodes have the ability to activate individual fascicles, but face challenges related to inflammation and scar tissue formation from direct contact of surrounding soft tissue (MacEwan et al., 2016; Wurth et al., 2017).

Similarly, surgical innovations such as targeted muscle reinnervation (TMR) (Dumanian et al., 2019), regenerative peripheral neural interfaces (RPNI) (Vu et al., 2018), and Agonist-antagonist Myoneural Interface (AMI) (Clites et al., 2018) have been developed to address these challenges and improve the balance between selectivity and stability. Although these methods have improved postamputation pain and motor functioning with prosthetics they fail to adequately address the problem of sensation (Hebert et al., 2014; Vu et al., 2018). The AMI has made strides in restoring proprioception, however, it is a complex surgical procedure that requires concurrent use with TMR or RPNI to restore motor function (Herr et al., 2021). Additionally, they are problematic from a mechanical and infectious standpoint with the use of surface electrodes in TMR and percutaneous wiring in RPNI (Schofield et al., 2020; Vu et al., 2020b). The clinical applications of these surgical techniques (Vu et al., 2020a; Herr et al., 2021; Karczewski et al., 2021) and electrode technology (Ghafoor et al., 2017; Rijnbeek et al., 2018; Larson and Meng, 2020; Raspopovic et al., 2020) has been reviewed in substantial detail in previous studies.

**FIGURE 1:**
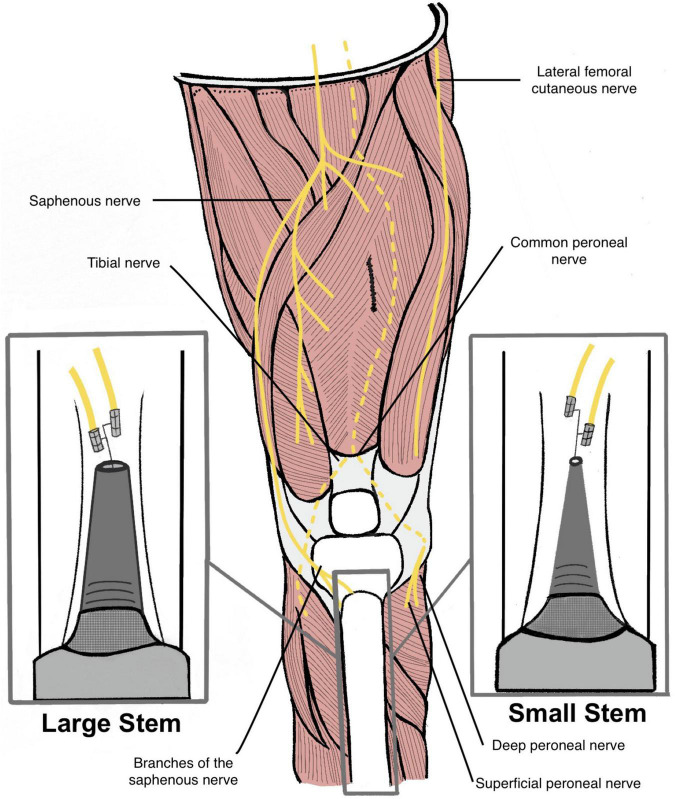
Illustration of a transtibial amputee with a large diameter stem endoprosthesis (left) and a small diameter stem endoprosthesis (right). Red line measurements indicate the different distances between the two endoprosthesis and the medullary canal (Not to scale). Cuff electrodes are wrapped around branches of the saphenous nerve and inserted into the medullary canal via cortectomy.

As an alternative solution to treating painful neuromas and achieving high fidelity signaling with long-term stability, we developed the Osseointegrated Neural Interface (ONI) (Figure 1; Israel et al., 2018; Dingle et al., 2020a). This technique combines the concepts of osseointegration and nerve regeneration to create a peripheral nerve interface that directly connects to an advanced prosthetic. It is based on the idea that the intramedullary canal can provide a protective environment that allows a nerve to regenerate and remain physiologically active (Dingle et al., 2020b). Beyond the clinical conceptualization of the ONI (Israel et al., 2018), experimental rabbit models have demonstrated that nerves transposed into bone are capable of transmitting both efferent (motor) and afferent (sensory) action potentials required for prosthetic control, including writing of sensory information into the CNS (Dingle et al., 2020a,b; Millevolte et al., 2021). Furthermore, regeneration of nerves transposed into the medullary canal has been demonstrated histologically (Dingle et al., 2020a), along with improvements in neural engagement as a product of time post amputation, as demonstrated in both cuff and sieve electrode types (Millevolte et al., 2021). Finally, in experimental models, osseointegration has served as a simple and stable solution for direct percutaneous connectivity (Dingle et al., 2020b; Millevolte et al., 2021). This provides a superior attachment for prosthetic limbs compare to the traditional socket given its mechanical stability, increased comfort, and improved range of motion (Ortiz-Catalan et al., 2014a). In many cases, clinical outcomes with osseointegration are equivalent if not better than the standard prosthetic socket and are also felt to provide a greater sense of embodiment (Al Muderis et al., 2018; Hoellwarth et al., 2020). Like TMR and RPNI, ONI is grounded in the surgical treatment of neuromas (Boldrey, 1943; Israel et al., 2018) that may serve as an innovative surgical option to optimize electrode implantation and percutaneous connectivity, toward improved prosthetic control.

The purpose of this anatomical study is to develop a preclinical model for surgical amputation and implementation of an ONI. While this is already a clinical technique used to treat neuromas, it has not yet been established clinically as a prosthetic interface. This study may encourage surgeons to approach amputations from a perspective that considers the peripheral nerves, their branching variations, and ultimately their ability to integrate an endoprosthesis that can house a neural interface at any amputation level. Integral to this study, we investigate the available volume within the medullary canal following osseointegration that may be utilized to construct a neural interface as part of an ONI.

## Materials and Methods

### Anatomic Dissection and Measurements

Dissection of the extremities is important for determining electrode design and guiding surgical implementation. The ONI concept has not yet been explored in humans and thus the available space in the medullary canal must be quantified at different amputation levels to determine how these electrodes can be incorporated. These parameters will dictate the size and configuration of the interface. In this study three upper extremity and three lower extremity cadaveric limbs were used for this study. One of the upper extremity limbs was a female donor while the rest of the limbs were male donors with the mean age being 67 years old (range 56–76 years old). Prior to this study the cadaver limbs had been used in other dissections. In the upper limbs the nerves identified and measured included the median nerve, ulnar nerve, and radial nerve. The lower extremity nerves included the common peroneal nerve (deep and superficial), tibial nerve, saphenous nerve, and medial and lateral sural nerves. Not all nerves were able to be identified in each specimen as some were cut during previous studies of these cadavers. During the dissection of the nerves, their corresponding branches and their target muscles were identified.

The circumference and depth from the skin surface of each nerve was measured at proximal, middle, and distal locations using a standard ruler. The elbow crease served as the proximal landmark and the proximal wrist crease as the distal landmark. In the lower extremity the proximal landmark was the fibular head, and the distal landmark was the lateral malleolus. For circumference, a small string was wrapped around the nerve and then measured using the ruler. These values are important for electrode design and safety protocol which relies on knowledge of neural anatomy. Nerve structure data in animal models varies significantly from human anatomical data and is therefore critical for clinical translation (Brill and Tyler, 2017). The length of the nerves was identified as the distance between the proximal elbow crease and distal wrist crease in the upper extremity and the proximal fibular head and the distal lateral malleolus in the lower extremity.

### CT Imaging Protocol

CT imaging of the specimens was obtained to evaluate and measure the long bones. This included the ulna and radius in the upper extremities and the tibia and fibula in the lower extremities. The length and medullary cavity diameter at proximal, middle, and distal locations was measured using the CT images. 3D software was then used for direct measurement of the medullary cavity volume, including the total medullary canal volume and diaphysis volume, at these locations in each bone. One of the specimens had a fibular fracture which prevented software measurement of the proximal fragment’s volume. This was calculated manually and added to the remaining fibular volume that could be measured by the software.

### Virtual Implantation and Contact

While most of the OI is performed in trans-femoral/humeral, transtibial amputations represent the largest number of amputations here in the United State. As such, our emphasis on transtibial, while also exploring transradial, is focused on delivering the greatest benefits to the amputee population in the United States (Haque et al., 2020). Additionally, a limitation in providing OI to transtibial amputees is the fluctuations in the medullary space, which we seek to address with our thin-stem OI implant. This is explored further in previous work focused solely on OI (Taylor et al., 2021).

**FIGURE 2:**
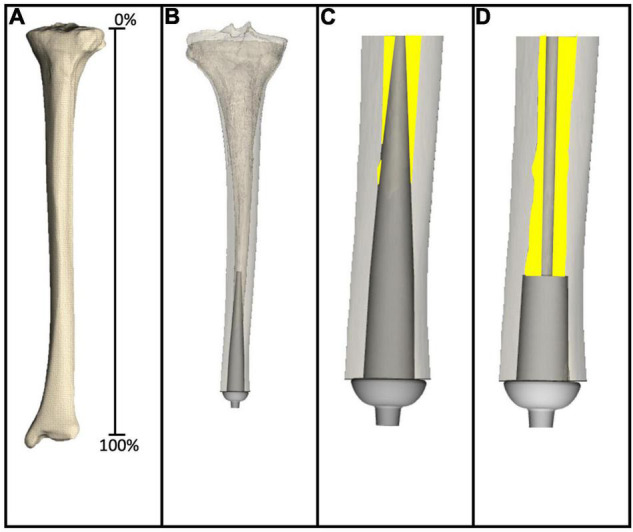
**(A)** Virtual tibia created from reconstructing CT scans to generate 3D computer model. **(B)** 3D computer model amputated to 80% residual length, and virtually implanted with a large diameter stemmed endoprosthesis. **(C)** Magnified image of the large diameter stemmed endoprosthesis virtually implanted into the virtual tibia, showing only the medullary canal analyzed in this study. Yellow highlights the remaining medullary canal available following virtual implantation. **(D)** Magnified image of the small diameter stemmed endoprosthesis virtually implanted into the virtual tibia. Yellow highlights the remaining medullary canal available following virtual implantation.

As detailed elsewhere (Taylor et al., 2021) virtual implantation techniques were developed and then used in this study. In review, axial CT scans were collected using a Siemens SOMATOM Definition Flash scanner, 120 kVp, 100 mAs, 512 × 512 acquisition matrix, 1-mm slice thickness, from one fully intact tibia. Using a desktop computer, the CT scans were reconstructed (MIMICS v21.0, Materialise, Plymouth, MI, United States) to generate a 3D model, which can be thought of as a virtual tibia (Figure 2A). Defining the residual as the distance between the average height of the tibial plateau and tibial plafond where percent length is measured proximal (0%) to distal (100%) (Taylor et al., 2020).

The medullary canal of one virtual tibia was prepared according to surgical instructions provided by a medical device manufacturer for POP/PODS systems (DJO Surgical, Austin, Texas) for the system usedin this example, including reamer, planar, and broach instrumentation as detailed by Drew et al. (2020). These steps dictated alignment and sizing of the endoprosthesis system, which were the target parameters to validate in this study. In this case, two example endoprosthetic designs were evaluated, both 10 cm in length from the collar to the proximal tip of the endoprosthesis. The large diameter stemmed endoprosthesis (Figures 2B,C) represented a traditional looking endoprosthesis with 3 cm of porous coating and 7 cm of stem, the majority of which was designed to be in direct contact with the medullary canal. The small diameter stemmed endoprosthesis (Figure 2D) also had 3 cm of porous coating, but the remaining 7 cm of stem was designed to avoid any contact with the medullary canal. Two primary questions of interest were addressed in this example: (1) How much difference is there in the percent bone removed to implant a small stemmed endoprosthesis when compared with a large stemmed endoprosthesis; and (2) how much medullary volume remains in the after implantation of the endoprosthesis for housing the nerves and electrodes required for an ONI.

The intact bone model was aligned to a global coordinate system defined by proximal anatomical landmarks (Taylor et al., 2020). 3D reconstructions of the amputated and surgically prepared bone were then globally aligned by surface fit to the intact tibia to reduce influence of alignment variation using the Global Registration function in 3-Matic (v13.0, Materialise, Plymouth, MI, United States). The virtually amputated reconstruction then underwent a virtual preparation procedure. Here, a computer CAD model matching the broach used in surgical preparation was coaxially aligned to the tibial shaft axis (Taylor et al., 2020) at the centroid of the distal medullary canal. The tibial shaft axis was defined by the inertial axis of the medullary canal from one medial-lateral proximal plateau width down the shaft (Taylor et al., 2020). The distal osteotomy plane was aligned perpendicular to this axis. This object was then subtracted from the 3D reconstruction to simulate the final surgical preparation of the bone.

For this analysis, fully intact CT scans of the bone were virtually implanted at six simulated amputation levels, specifically, 30, 40, 50, 60, 70, and 80% residual length. The metaphyseal regions were excluded as this would either not support the stack-up of an exoprosthetic limb above the ankle at the distal end, nor would not allow for knee function in very short residual lengths (Taylor et al., 2020).

### Virtual Implantation Data Analysis

Through virtual implantation the remaining residual medullary volume was calculated to help determine the amount of space available for the ONI. The percent of medullary bone removed during virtual implantation was calculated using surface and volume measurements in 3-Matic. Bone removed was the volume of bone intersecting with the endoprosthesis as a percent of total volume of the original region of interest. The volume of remaining medullary canal after virtual implantation was calculated using in 3-Matic.

## Results

### Nerve Identification and Measurements

The nerve measurements provide important information for the incorporation of a neural interface that requires implantation of electrodes around or into the major peripheral nerves. With an ONI design, the nerve(s) must be able to reach the electrodes within the bone with sufficient length and medullary space. The major peripheral nerves in the upper extremity evaluated in this study included the median nerve, ulnar nerve, and radial nerve. In the lower extremity six nerves were measured including the tibial nerve, superficial peroneal nerve, deep peroneal nerve, lateral and medial sural nerve, and the saphenous nerve. All nerves were measured at proximal, middle, and distal locations (Tables 1, 2). Additional measurements included the sensory branch of the ulnar nerve and branches of the tibial nerve. The following nerves were unidentifiable: Lateral sural (*n* = 1), Medial Sural (*n* = 1) as listed in Table 2. The following segments of nerves were unidentifiable: Distal portions of the superficial peroneal nerve (*n* = 1), Saphenous nerve (*n* = 3), and the radial nerve (*n* = 1). Proximal and middle portions of the lateral antebrachial cutaneous nerve (*n* = 1). In the upper extremity the median nerve circumference was the largest with a mean of. 13.17 ± 1.79 mm while the mean ulnar nerve and radial nerve circumference was 10.33 ± 0.67 and 9.10 ± 2.94 mm, respectively. The sensory branch of the ulnar nerve had the smallest circumference of all ulnar nerve measurements (Table 1). In the upper extremity, the tibial nerve had the largest circumference with a mean of 13.67 ± 3.88 mm. The common peroneal nerve was measured after splitting into superficial and deep branches which had similar mean circumferences, 8.50 ± 0.27 and 8.28 ± 1.09 mm, respectively. The sensory nerves had the smallest mean circumferences with the lateral sural nerve measuring 8.00 ± 2.01 mm, the medial sural nerve measuring 7.42 ± 0.89 mm, and the saphenous nerve measuring 5.50 ± 1.18 mm (Table 2). In addition to circumference, the depth of each nerve from the surface of the skin was measured and is useful for determining the extent of anatomical dissection require to reach each nerve. The radial nerve and the tibial nerve had the largest depths in each extremity (Tables 1, 2).

**TABLE 1:** Upper extremity nerve measurements.

	Circumference (mm)			Depth (mm)		
		
Nerve	#1	#2	#3	#1	#2	#3
**Ulnar nerve**						
Proximal	18.0	13.0	15.0	14.0	15.0	23.0
Middle	10.5	9.0	11.0	16.0	14.0	15.0
Distal	10.5	8.0	10.0	8.0	9.0	7.0
Sensory branch	7.5	5.0	6.5	−	−	−
**Median nerve**						
Proximal	19.5	13.0	14.0	21.0	25.0	27.0
Middle	15.0	8.5	12.0	28.0	18.0	18.0
Distal	12.0	12.0	12.5	9.0	7.0	7.0
**Radial nerve**						
Proximal	12.0	12.0	13.0	22.0	22.0	27.0
Middle	8.5	14.0	10.0	32.0	22.0	30.0
Distal*	N/A^δ^	5.0, 6.0	7.5, 8.0	N/A^δ^	5.0	5.0
**LACN^φ^**						
Proximal	13.0	N/A^δ^	8.5	−	−	−
Middle	8.5	N/A^δ^	6.0	−	−	−

*Branch to extensor side, branch to thumb. ^δ^Unable to measure. ^φ^LACN, Lateral Antebrachial Cutaneous Nerve. -depth not measured for cutaneous nerves.

**TABLE 2:** Lower extremity nerve measurements.

	Circumference (mm)			Depth (mm)		
		
Nerve	#1	#2	#3	#1	#2	#3
**Tibial nerve**						
Proximal	20.0	17.0	16.0	25.0	45.0	27.0
Middle	14.0	16.5	12.5	35.0	42.0	32.0
Distal	14.0	21.0	18.0	10.0	12.0	5.0
Popliteal fossa	13.0	13.0	16.5	20.0	28.0	24.0
Branch to medial gastrocnemius	8.5	12.0	5.0			
Branch to Soleus	7.0	11.0	11.0			
**Superficial peroneal nerve**						
Proximal	9.0	11.5	11.0	8.0	10.0	7.0
Middle	8.0	8.5	6.0	5.0	8.0	7.0
Distal	6.5	8.0	8.0	6.0	8.0	3.0
**Deep peroneal nerve**						
Proximal	13.5	9.0	10.0	8.0	10.0	7.0
Middle	6.0	6.0	5.5	18.0	28.0	25.0
Distal	9.0	9.5	6.0	10.0	10.0	7.0
**Lateral sural nerve**						
Proximal	5.5	12.0	N/A	4.0	4.5	N/A
Middle	5.5	8.0	N/A	5.0	8.0	N/A
Distal	8.0	9.0	N/A	2.5	9.0	N/A
**Medial sural nerve**						
Proximal	6.5	N/A	9.0	6.0	N/A	10.0
Middle	6.0	N/A	7.0	5.0	N/A	7.0
Distal	8.0	N/A	8.0	2.5	N/A	4.0
**Saphenous nerve**						
Proximal	5.5	6.5	11.0	7.0	12.0	27.0
Middle	3.5	4.5	2.0	4.0	7.0	3.0
Distal	N/A	N/A	N/A	N/A	N/A	N/A

*N/A, unable to measure.*

### CT Imaging and Long Bone Measurements

The long bones of the upper and lower extremity were measured on CT imaging to analyze medullary cavity volume and diameter and proximal, middle, and distal locations. This information is important for quantifying the space available for an ONI at a given amputation level. In the upper extremity the mean medullary cavity volume of the ulna and radius was 11.15 ± 1.70 and 13.30 ± 3.84 mL and mean length was 247.0 ± 11.27 and 226.7 ± 7.57 mm, respectively. Mean medullary cavity diameter at the midpoint was 5.07 × 4.43 ± 0.76 × 0.75 mm for the ulna and 6.10 × 4.13 ± 1.21 × 0.12 mm for the radius (Table 3). In the lower extremity the mean medullary cavity volume of the tibia and fibula was 180.92 ± 13.45 and 23.29 ± 12.44 mL and the mean length was 376 ± 13.23 and 378.67 ± 14.89 mm. Mean medullary cavity diameter at the midpoint was 13.73 × 12.10 ±. 2.23 × 1.04 mm for the tibia and 7.00 × 5.10 ± 1.28 × 0.81 mm for the fibula (Table 4). The variation in measurements is representative of the differing morphologies among the long bones.

**TABLE 3:** Upper extremity bones.

	#1	#2	#3
Age/Gender	76 M/L	57 F/R	76 M/R
Ulna length (mm)	253	234	254
**Ulna medullary cavity diameter (mm)**			
Proximal	8.3 × 6.2	8.5 × 6.6	9.1 × 7.1
Middle	4.4 × 5.2	4.9 × 3.7	5.9 × 4.4
Distal	5.1 × 4.2	5.8 × 5.1	6.2 × 4.2
Ulna total medullary cavity volume (mL)	9.8	10.59	13.06
Ulna diaphysis medullary cavity volume (mL)	3.23	3.41	4.72
Radius length (mm)	230	218	232
**Radius medullary cavity diameter (mm)**			
Proximal	9.7 × 8.0	6.5 × 6.4	9.3 × 8.7
Middle	6.8 × 4.0	4.7 × 4.2	6.8 × 4.2
Distal	8.4 × 4.6	7.2 × 5.3	10.0 × 5.9
Radius total medullary cavity volume (mL)	14.72	8.95	16.22
Radius diaphysis medullary cavity volume (mL)	7.6	5.24	7.86

**TABLE 4:** Lower extremity bones.

	#1	#2	#3
Age/Gender/Right or Left	73 M/L	56 M/R	66 M/L
Tibia Length (mm)	386	381	361
**Tibia medullary cavity diameter (mm)**			
Proximal	22.0 × 20.3	27.7 × 21.2	23.1 x. 19.7
Middle	11.7 × 10.9	13.4 × 12.8	16.1 × 12.6
Distal	13.1 × 12.1	14.5 × 14.1	16.6 × 14.0
Tibia total medullary cavity volume (mL)	184.16	166.15	192.46
Tibia diaphysis medullary cavity volume (mL)	41.66	60.36	71.09
Fibula length (mm)	396	383*	357*
**Fibula medullary cavity diameter (mm)**			
Proximal	6.0 × 2.9	8.4 × 4.9	6.8 × 5.2
Middle	5.6 × 5.3	7.3 × 5.8	8.1 × 4.2
Distal	6.1 × 3.5	8.0 × 5.1	8.5 × 4.5
Fibula total medullary cavity volume (mL)	25.18	34.68	10.01**
Fibula diaphysis medullary cavity volume (mL)	3.41	9.33	4.59**

*Segmental fibular fracture. **Proximal Fibular measurement excluded.

### Virtual Implantation and Medullary Canal Volume

The data from the virtual implantation provided basic information for incorporation of the ONI and electrodes in the medullary canal space. Results revealed that the large diameter stem design required considerably more bone removal than the small diameter stem design, especially at the longer residual lengths (Table 5). At 50, 60, 70, and 80% residual length, the small diameter stem designed removed 20, 32, 44, and 46% less bone, respectively, from the medullary canal than the large diameter stem design (Table 5). Concentrating on the volume of the medullary canal available for ONI at the various residual lengths, there is a notable decrease in available volume, independent of stem diameter, as the length of the residual limb increases (Table 6). At 30% residual, the large diameter stem has 101,552 mm^3^ of space available between the medullary surface and the endoprosthesis. As the residual limb length increases to 80%, the volume available decreases to only 2,857 mm^3^. Likewise, at 30% residual, for example, the small diameter stem has 107,505 mm^3^ of space available between the medullary surface and the endoprosthesis. As the residual limb length increases to 80%, the volume available decreases to only 5,112 mm^3^—clearly tied to the anatomical shape of the medullary canal.

**TABLE 5:** Percent of medullary bone removed for virtual implantation.

Residual length (%)	% Removed from medullary canal surrounding large diameter stem	% Removed from medullary canal surrounding small diameter stem	Difference (%)
30	0.82	0.82	0%
40	0.49	0.48	−2%
50	0.50	0.40	−20%
60	0.64	0.43	−32%
70	0.92	0.51	−44%
	1.78	0.97	−46%

**TABLE 6:** Volume of remaining medullary canal after virtual implantation.

Residual length (%)	Remaining MC volume after large diameter stem implantation (mm^3^)	Remaining MC volume after small diameter stem implantation (mm^3^)	Difference between small diameter stem and large diameter stem (mm^3^)	Change between large diameter and small diameter stem (%)
30	101,552	107,505	5,953	6%
40	30,532	33,457	2,925	9%
50	11,573	13,919	2,346	17%
60	6,591	8,362	1,771	21%
70	4,866	6,298	1,431	23%
80	2,857	5,112	2,256	44%

## Discussion

While the ONI is a clinically demonstrated treatment for neuropathic pain (Israel et al., 2018) and there is a wealth of evidence on osseointegration, the combination of these two concepts in humans remains unknown (Al Muderis et al., 2018; Hoellwarth et al., 2020; Overmann et al., 2020), In experimental rabbit models, it has been demonstrated that physiologic function of the transposed nerve is maintained with evidence of morphological and physiological stability, including recorded signals (Dingle et al., 2020b). A widely accepted trade-off for peripheral nerve interfaces is that of selectivity and invasiveness and their impact on chronic stability. It is well established that the most selective interfaces are also the most invasive in nature and provide the least stability and longevity. In a proof-of-concept experiment in rabbits, the ONI has demonstrated its capability to stably house sieve electrodes, typically regarded as the most selective but also invasive of sophisticated electrodes. Through utilization of bone to create a protective environment, the ONI was capable of recording compound nerve action potentials (CNAPs) that improved over time in addition to generating somatosensory evoked potentials (SSEPs) from a greater number of channels (Millevolte et al., 2021). This alteration to the surgical approach as opposed to the engineering approach may provide valuable stability and longevity to the more selective types of peripheral nerve interfaces. Additionally, the use of osseointegration provides mechanical stability and thereby mitigating the safety and reliability issues seen with percutaneous leads in other interface designs (Ortiz-Catalan et al., 2014b).

The results in this study help lay the groundwork for a pre-clinical ONI design. Dissection and identification of the major peripheral nerves is critical in determining how they will be incorporated into a bidirectional neural interface. Depending on the level of amputation and/or goals of a patient the number and size of electrodes and corresponding nerves used in the interface will vary. An individual with a more proximal amputation may require more electrodes and use more nerves or nerve branches compared to someone with a more distal amputation. The data presented herein demonstrates the variability in nerve circumference and branching along the length of the nerve. The circumference measurements of the upper extremity demonstrate trends similar to what is seen in the current literature. Circumference decreases distally, likely representing the increased branching that occurs, with the exception of the median nerve having an increase most distally as it enters the carpal tunnel (Bargalló et al., 2010; Delgado-Martínez et al., 2016; Brill and Tyler, 2017). These values will play a critical role in electrode design and clinical application. While non-invasive methods such as MRI or ultrasound are available for quantifying nerve area and ratio, they are not sufficient for assessing more detailed morphology (Brill and Tyler, 2017). These nerve dimensions are important for determining the appropriate size of different types of electrodes. A study by Tan et al. (2014) outlined these specifics for flat interface nerve electrodes (FINEs) and spiral electrodes which utilized a 10 mm opening size for median and ulnar nerves and a 4 mm diameter, respectively. These dimension, in conjunction with knowledge of structural and dynamic peripheral nerve anatomy, guide construction of these electrodes. The same type of electrode will demonstrate varying levels of selectivity depending on which nerve they are used on. For example, a FINE electrode has higher selectivity when placed on the femoral nerve compared to the tibial and common peroneal nerves (Schiefer et al., 2013). This is influenced by fascicular anatomy as well as the location of electrode placement along the nerve. While animal models are useful in mapping out function for different electrodes, their peripheral nerve anatomy is vastly different than humans and therefore human anatomic studies are necessary for clinical translation.

Additionally, an individual seeking sophisticated control with restoration of sensation will likely require a more extensive neural interface design that necessitates the need for more medullary space. While other surgical techniques, such as TMR and RPNI, have demonstrated success with motor control, they have struggled to adequately restore sensation (Santosa et al., 2020). The ONI offers an alternative solution that can also be used to compliment current motor control techniques and serves as the interface dealing solely with sensory input. To achieve this, an endoprosthesis must be incorporated into the long bone while the neural interface components are housed in the residual medullary space. In doing so, one must consider the distance of the nerve from the bone, leaving adequate length to transpose the nerve without tension, which in some cases may require nerve preserved beyond the length of the residual stump. Strategic surgical planning is necessary for optimizing the amount of space provided by the medullary canal. A nerve(s) and its corresponding electrode must be small enough to fit within the allocated space. For example, in this study the average diameter of the proximal medullary canal of the ulna and radius are 8.6 × 6.6 mm and 8.5 × 7.7 mm, respectively. The average diameter, as estimated from the circumference, of the ulnar nerve and radial nerve are 3.3 and 2.1 mm, respectively. Given that the standard spiral cuff electrodes are “self-sizing” and fit to match the nerve’s diameter, these medullary dimensions are large enough to house these electrodes (Christie et al., 2017). The nerve and medullary space measurements vary between proximal, middle, and distal locations (Tables 1–4). These values must be taken into consideration when determining location of electrode placement along a nerve and how that fits into a corresponding amputation level. Furthermore, these electrodes must be designed to maximize SNR of neural signals while minimizing nerve compression and cross talk from neighboring muscles (Raspopovic et al., 2020). Although electrodes with more complex configurations are able to achieve higher selectivity, their use can be limited by physical space and subsequent damage from the surrounding environment (Sabetian and Yoo, 2017). In contrast, the medullary canal offers a more protective space for these complex figurations by decreasing electrical cross talk and nerve compression from nearby muscles. The mechanical stability of bone reduces this interference by providing electrical and physical insulation (Millevolte et al., 2021). This could potentially improve the spatial selectivity of extraneural electrodes and increase the longevity of intraneural electrodes.

Residual medullary space is dependent on endoprosthetic stem design, which can be demonstrated by quantifying the amount of space available in the long bones of the upper and lower extremity through CT imaging and virtual implantation. The virtual implantation data demonstrates the difference between large stem and small stem endoprosthetics and the amount of residual bone available at varying amputation levels. Clinically, this is important when the quantity and quality of existing bone in the residual limb is limited. Mechanical stability is dependent on maximizing the contact area between the endosteum and the osseointegration region (Drew et al., 2020). However, this must be balanced with the need to maintain sufficient cortical support which is dictated by the amount of bone removed for osseointegration. Additionally, quantification of the residual medullary space proximal to an osseointegration can help determine how much space is available for the housing of a neural interface with implantable electrodes. Comparing the results, it is not surprising that the small diameter stem design consistently had more medullary canal volume available when compared to the large diameter stem design. Thinner stems require less cortical bone be reamed/removed from the medullary canal in order to insert the OI (Taylor et al., 2021). Thin stem allows for more intact, and therefore stronger bone with no loss of integral bone anchoring region. In addition to retaining more of the native bone and therefore its integrity, it also provides more space in which to house delicate nerves and neural interfacing materials. While the purpose of these data is not to indicate a specific stem geometry, it is helpful to understand what space could be made available with such a simple design change. A critical component of the ONI design involves maximizing endoprosthetic stability while maintaining enough residual medullary space to incorporate a neural interface and its accompanying hardware.

## Conclusion

The measurements obtained in this study provide a framework that can guide surgical implementation of an ONI. Identification of nerve length patterns allows for optimization of neural interfacing electrodes and prosthesis requirements at a given amputation level. Morphologic bone evaluation and virtual implantation data provides information required for successful integration of intramedullary electrodes with an endoprosthesis. The data collected in this study will be used to build ONIs that maximize neural interfacing and osseointegration stability with optimized residual space without compromising load distribution at different amputation levels.

## Data Availability Statement

The original contributions presented in the study are included in the article/supplementary material, further inquiries can be directed to the corresponding author/s.

## Author Contributions

AK participated in concept development, carried out the experiment, and prepared the manuscript. WZ involved in experimental design and data collection. LS helped carry out the experiment and was involved in image analysis. KB designed virtual implantation model, computational framework, and analyzed the data. AD and SP supervised the project and contributed to the final version of the manuscript. All authors contributed to the article and approved the submitted version.

## Conflict of Interest

The authors declare that the research was conducted in the absence of any commercial or financial relationships that could be construed as a potential conflict of interest.

## Publisher’s Note

All claims expressed in this article are solely those of the authors and do not necessarily represent those of their affiliated organizations, or those of the publisher, the editors and the reviewers. Any product that may be evaluated in this article, or claim that may be made by its manufacturer, is not guaranteed or endorsed by the publisher.
